# Subthalamic Neurons Encode Both Single- and Multi-Limb Movements in Parkinson’s Disease Patients

**DOI:** 10.1038/srep42467

**Published:** 2017-02-13

**Authors:** Ariel Tankus, Ido Strauss, Tanya Gurevich, Anat Mirelman, Nir Giladi, Itzhak Fried, Jeffrey M. Hausdorff

**Affiliations:** 1 Center for study of Movement, Cognition and Mobility, Department of Neurology, Tel Aviv Sourasky Medical Center, Tel-Aviv 6423906, Israel; 2 Functional Neurosurgery Unit, Tel Aviv Sourasky Medical Center, Tel Aviv 6423906, Israel; 3 Department of Neurology and Neurosurgery, Sackler Faculty of Medicine, Tel Aviv University, Tel Aviv 6997801, Israel; 4 Sagol School of Neuroscience, Tel Aviv University, Tel Aviv 6997801, Israel; 5 Department of Neurology, Tel Aviv Sourasky Medical Center, Tel-Aviv 6423906, Israel; 6 Sieratzki Chair in Neurology, Sackler School of Medicine, Tel Aviv University, Tel Aviv 6997801, Israel; 7 Department of Neurosurgery, University of California, Los Angeles, CA 90095, USA; 8 Department of Physical Therapy, Sackler Faculty of Medicine, Tel Aviv University, Tel Aviv 6997801, Israel

## Abstract

The subthalamic nucleus (STN) is the main target for neurosurgical treatment of motor signs of Parkinson’s disease (PD). Despite the therapeutic effect on both upper and lower extremities, its role in motor control and coordination and its changes in Parkinson’s disease are not fully clear. We intraoperatively recorded single unit activity in ten patients with PD who performed repetitive feet or hand movements while undergoing implantation of a deep brain stimulator. We found both distinct and overlapping representations of upper and lower extremity movement kinematics in subthalamic units and observed evidence for re-routing to a multi-limb representation that participates in limb coordination. The well-known subthalamic somatotopy showed a large overlap of feet and hand representations in the PD patients. This overlap and excessive amounts of kinematics or coordination units may reflect pathophysiology or compensatory mechanisms. Our findings thus explain, at the single neuron level, the important subthalamic role in motor control and coordination and indicate the effect of PD on the neuronal representation of movement.

Neurons in the subthalamic nucleus (STN) are known to encode motor information. Examples include hand grip force^1^, the timing of target appearance or hand movement onset and movement direction^2^, and tremor in Parkinson’s disease patients^3^. Patients with Parkinson’s disease who performed alternating bimanual movements showed reduced fMRI activity in the basal ganglia, compared with healthy subjects^4^. In addition to hand movements, STN deep brain stimulation may ameliorate gait and postural symptoms of Parkinson’s disease^5^,^6^. Recently, single subthalamic neurons were observed to modulate voluntary movements, with little activity during imagery of gait, suggesting that the STN controls movement execution that is not likely to be gait-specific^7^. In the monkey, STN activity has been related to movement direction, amplitude and velocity^8^, with 28% of the cells firing in relation to active arm movements, 15% to leg movements, and 18% to orofacial movements^9^.

The STN has also been implicated in *motor coordination* during movement initiation or suppression^10^. Its electrical stimulation was shown to have different effects on force application during bimanual and unimanual grasping^11^,^12^,^13^. It normalizes gait coordination^14^, improves coordination of hand preshaping^15^ and enhances gait symmetry^16^, all of which are required for gait coordination. In animals with Parkinson’s disease, changes in subthalamic activity patterns were accompanied by deficits in motor coordination^17^. In patients with traumatic brain injury^18^ and older adults^19^, STN was activated less during motor coordination relative to younger healthy controls.

Despite the few aforementioned studies, the precise involvement of single subthalamic cells in motor coordination in patients with Parkinson’s disease and the exact type of motor information they encode are still largely unknown. The goal of our study was to characterize the encoding of motor information in single neurons in the STN of patients with Parkinson’s disease, and the influence of the disease on the representation. In particular, we focused on movement kinematics and participation in bipedal or bimanual coordination.

## Results

We recorded the activity of 89 units in the STN of ten Parkinson’s disease patients intra-operatively (Table 1).

### Direct Neuronal Encoding of Kinematics

**Figure 1:**
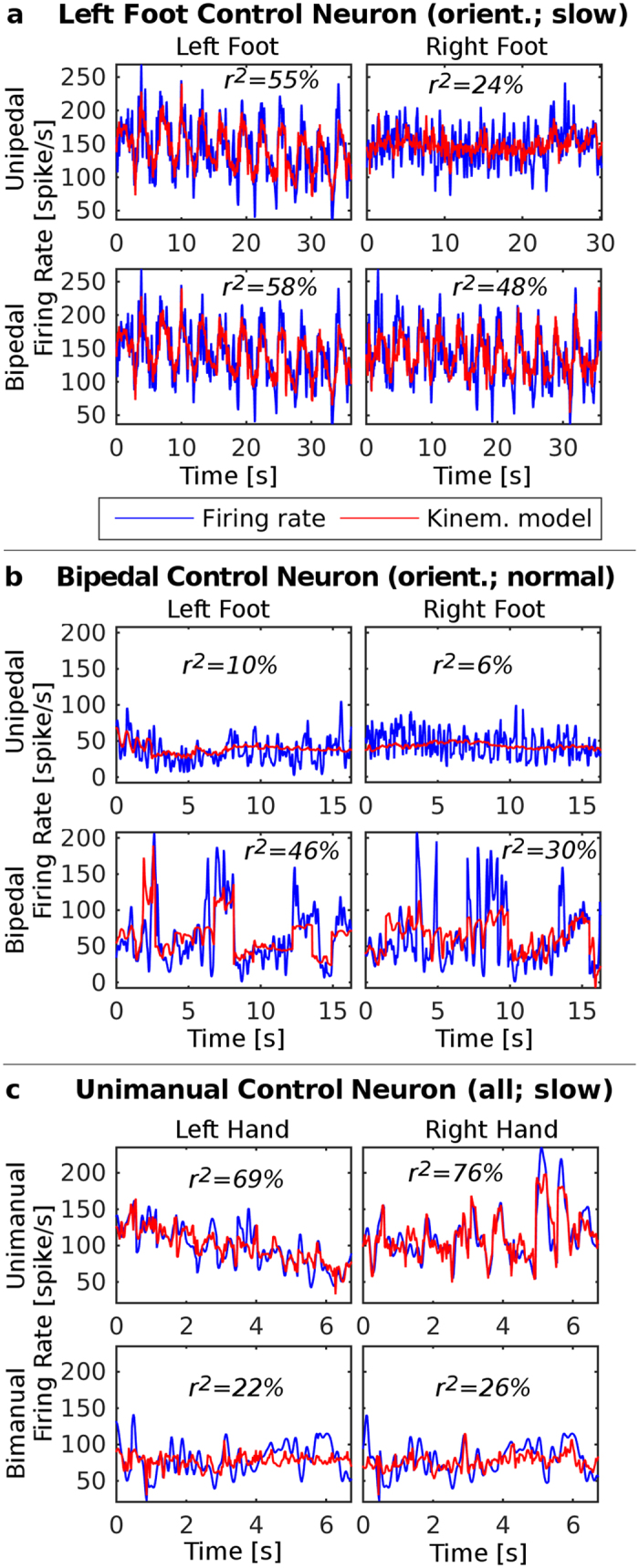
Three right STN neurons, each demonstrating a different type of selectivity. Each graph shows the smoothed firing rate (blue) and the corresponding linear regression kinematic model (red) at a certain condition, along with the coefficient of determination (r^2^) between them (see Methods). In parentheses above each graph are the kinematic parameters composing the model, and the pace of movements or performing limb employed during this condition. (**a**) The neuron is responsive to left foot movements during both the unipedal and bipedal conditions. It is also related to orientation of the right foot during bipedal movements, but this may be due to the high correlation between orientation of the left and right feet. The correlation is much lower for unipedal right foot movements. (**b**) Bipedal control neuron. The firing rate is related to normal-pace orientation of either foot during bipedal movements, but not during unipedal movements. (**c**) Unimanual control neuron. A combined model of orientation, angular velocity, and acceleration during slow movements explains high percentages of the variability in firing rate during unimanual, but not bimanual, movements.

To directly evaluate the neuronal code, we modeled the firing rate function by linear models based solely on kinematics: orientation, angular velocity, and acceleration (see Methods). Figure 1 displays the firing rates of three example neurons along with their linear estimation models. For a neuron to be considered “directly encoding kinematics”, we required two criteria: the correlation between its model and firing rate is significant (see Methods) and the model explains a considerable percentage of the variability of the firing rate (coefficient of determination: *r*^2^ > 0.30). Based on these two criteria, 43% (38/89) of the recorded units directly encoded movement kinematics. This percentage is comparable to the one reported in the literature for passive movements (49%)^20^.

**Figure 2:**
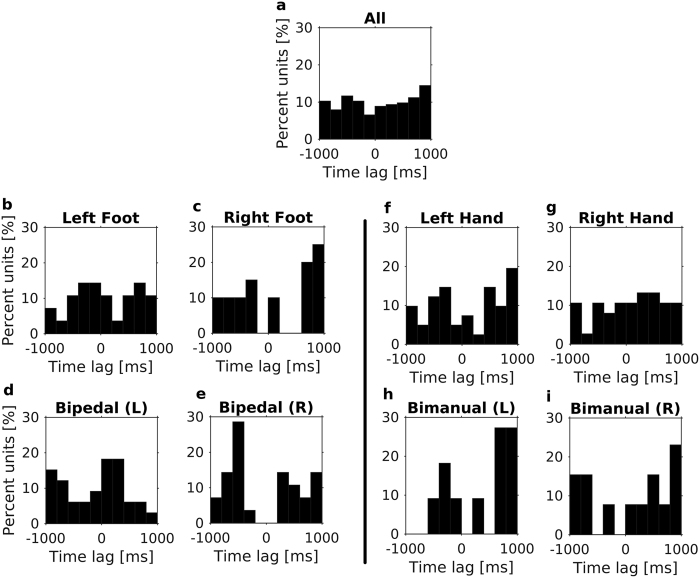
Histograms of the optimal time lags used in the linear models mapping kinematics to firing rates. (**a**) The percentage of models for each range of time lags. Models include all types of movement. **(b–i)** The same as (**a**), but only models for movements of a specific limb in either a single-limb or a two-limb movement are included. Above each histogram is the type of movement. For two-limb movements, the parentheses (L/R) denote whether kinematics of left or right limb was employed in the model, respectively. For all types of movement, both positive and negative optimal time lags were observed. The large range of optimal time lags may result from Parkinson’s disease.

The time lags between the neuronal firing and movement kinematics were almost uniformly distributed in the examined range between (−1)s and 1s (Fig. 2a). The optimal time lags of models generated for each type of movement (unipedal/bipedal/unimanual/bimanual) also expand the whole range (Fig. 2(b–i)). For all types of movement, there were units whose firing preceded the movement itself, whereas for others, it succeeded the movement. Similarly, when examining the different types of kinematic models (i.e., models based on orientation, angular velocity, acceleration or the combination of the three), time lags showed large variations. As discussed below, this lack of specificity may be due to Parkinson’s disease.

### Neurons Containing Kinematic Information

Do subthalamic neurons also utilize other, indirect, maybe non-linear, encoding schemes to represent kinematic information? We investigated this question using the entropy correlation coefficient, an information-based measure of dependence^21^, and found significant relations between firing rate and kinematics in 93% (83/89) of recorded units in at least one condition (Bonferroni corrected). All 83 responsive units were significantly related to all three kinematic parameters: orientation, angular velocity, and acceleration in a manner that was independent of the other two parameters (conditional entropy correlation coefficient) in at least one condition. This indicates that almost all of the recorded units encoded kinematic information in their firing rate.

### Motor Coordination

Bimanual coordination dysfunction is a sign of Parkinson’s disease^22^,^23^. We therefore first compared whether patients performed movements of two limbs and of a single limb at a similar pace. The duration of the two types of movement (coordinated vs. single-limb) performed by the same limb did not differ significantly (paired-sample t-test, *p* > 0.06 in all 12 tests: all paces (slow, normal, fast), examined for each of the 4 limbs).

Most of the recorded units (78%; 69/89) were related to motor control of *both limbs*, where movement of the other (non-encoded) limb either activated the neuron (i.e., units responsive during bipedal or bimanual movements, but *not* during unipedal or unimanual ones), or deactivated it (i.e., units responsive during unipedal or unimanual movements, but *not* during bipedal or bimanual ones). We refer to the former as “pure bipedal” or “pure bimanual” units, and to the latter as “pure unipedal” or “pure unimanual” units. An example of a pure bipedal neuron appears in Fig. 1b, where significant relationships between the firing rate and foot orientation can be established only during bipedal movements, but not during unipedal ones. Figure 1c exhibits a pure unimanual neuron, whose firing and kinematics are strongly correlated during the unimanual movements, but not during the bimanual ones. Only a minority of 16% (14/89) of the recorded units were not affected by the other limb. They represented kinematics of the same limb independently of whether or not the other limb participated in the movement (i.e., during both uni- and bi-pedal movements or during both uni- and bi-manual ones; see Fig. 1a).

The population of pure bipedal and pure bimanual units composed of 27% (24/89) of recorded STN units, with 11% (10/89) pure bipedal units, and 22% (20/89) pure bimanual units. The entropy cross correlation of units in this population was significant during bipedal or bimanual tapping, but *not* during any of the two unipedal or two unimanual conditions. As these findings suggest, in a subpopulation of 7% (6/89) of the recorded units, each unit was related to both bipedal *and* bimanual movements, but not to any movement of one limb by itself, indicating involvement in left-right limb coordination in both upper *and* lower extremities. The proportion of this population agreed with random mixture of the pure bipedal with the pure bimanual properties (see Methods; Chi-square test of homogeneity, p = 0.17, χ^2^ = 1.90, 1 degree of freedom).

More than half the recorded neurons (51%) encoded the kinematics of single-limb movements only, but not during bipedal or bimanual movements. Table 2 presents the segmentation of this population according to the laterality of the related limb: contralateral, ipsilateral or units related to both ipsi- and contra-lateral single-limb movements, and to the type of extremity: upper, lower, or related to single-limb movements of both upper and lower extremities. The percentage of units in the latter subpopulation (Table 2, row 3) matches a random mixture of encoding of feet and hand movements (Chi-square test of homogeneity, p = 0.53, χ^2^ = 0.39, 1 degree of freedom; see Methods). Thus, our recordings do not lend support to a significant subthalamic abstraction of whether the acting limb is upper or lower. In contrast, the percentage of units related to both contra- and ipsi-lateral movements (Table 2, Column 3) was significantly higher than would be expected by a random mixture of properties in the population (Chi-square test of homogeneity, p = 0.04, χ^2^ = 4.44, 1 degree of freedom; see Methods). Our findings thus indicate that subthalamic units encode movement kinematics independently of the laterality of the performing limb, and similarly for single-limb hand movements. Some units represented 1-foot movements (independently of the acting foot), and similarly, some units encoded 1-hand kinematics.

### Localization of Neurons Representing Kinematics and Somatotopy

**Figure 3:**
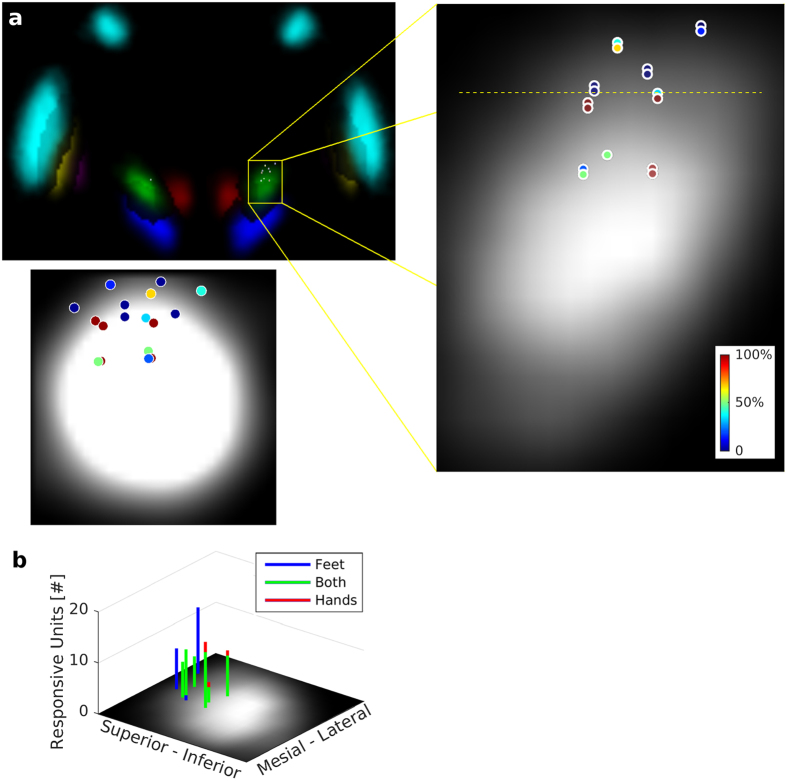
Localization of task-related neurons and somatotopy. (**a**) Recording sites marked on a coronal section of a 7T MRI subcortical probabilistic atlas^45^, 15 mm posterior to AC (STN: green; substantia nigra: blue; red nucleus: red; GPi: magenta; GPe: yellow; Striatum: cyan). In the expanded image, the gray background indicates the STN. Similarly, the bottom panel is a sagittal section 12 mm from the midline showing the STN as gray levels. In both sections, the location of each electrode is marked by a circle and colored according to the percentage of units recorded on that electrode which directly encode movement kinematics (see Results). Higher percentages of such units were recorded by electrodes inferior to the yellow dashed line, which is 3.6 mm below the AC-PC line. (**b**) Subthalamic somatotopic organization with overlap in the representation of feet and hand movements. The amount of units related to feet movements only (blue), hand movements only (red), and both hand movements and feet movements (green) is displayed as a stacked histogram plotted at recording locations. Units related solely to feet movements were found in the superior-mesial part only, whereas those related merely to hand movements lied in the more inferior-lateral part of the recording zone. The figure shows only 8 recording sites, because it depicts the results for the 9 patients implanted in the Right STN, and for one of these subjects this recording site did not yield any responsive units.

To localize sub-areas of the STN with high concentration of units directly encoding kinematics, we examined for each electrode the percentage of units it recorded which directly encode movement kinematics (Fig. 3a). We found that the more inferior electrodes, at least 3.6 mm below the AC-PC line, recorded significantly higher percentages of kinematics-encoding neurons (mean = 64%, SE = 11%) in comparison with those in the superior part of the recorded area (less than 3.6 mm below the AC-PC line; mean = 15%, SE = 9%; two-sample right-tailed t-test: *p* = 0.0027). Note, that due to operating room constraints which prevented an exhaustive search, the most inferior electrode sampled was 5.4 mm below the AC-PC line.

Over a quarter of the recorded units (27%; 24/89) held kinematic information related to feet movements only (i.e., *not* to hand movements), whereas only 4% (4/89) were solely related to hand movements (i.e., *not* feet movements). The proportions of the two populations were significantly different (Chi-square test of homogeneity, p < 0.0001, χ^2^ = 17.0, 1 degree of freedom). These populations were organized somatotopically within the superior 40% of the STN, with feet movements at the ankle represented more superior-mesially, and hand movements at the wrist, more inferior-laterally (Fig. 3b). Notwithstanding, almost two thirds (62%; 55/89) of the recorded units were related to both feet movements and hand movements. This large overlap in representation was also located somatotopically (Fig. 3b).

## Discussion

We identified units in the STN of patients with Parkinson’s disease representing movement kinematics in a complex manner that depends on the limb performing the movement. The vast majority of recorded units were affected by the movement, or lack of movement, of both limbs. We identified a population of STN units related to the control of bipedal and bimanual movements which was not involved in controlling the corresponding unipedal or unimanual movements. A complementary subthalamic population participated in the control of unipedal or unimanual movements, but not of bipedal or bimanual ones. We observed somatotopic organization of the STN, with the ankle represented more superior-mesially, and the wrist, inferior-laterally, and with a large overlap between the two.

In the neurologically-intact STN of the monkey, the responses of 91% of the responding cells were elicited by manipulations around a single joint only^24^. That study indicates a high degree of limb specificity in the responsiveness of normal STN neurons. When comparing this with our findings of a large neuronal population responsive to multi-limb movements, assuming similarity between humans and monkeys, we infer that the excessive amount of multi-limb-related units may be a result of either pathophysiology due to Parkinson’s disease, or a compensatory mechanism that re-routes motor coordination function to the STN. The decrease in specificity of the neuronal response in the STN may also be related to the decrease in accuracy of movements of patients with Parkinson’s disease (i.e., higher variability of movement endpoints)^25^.

In patients with Parkinson’s disease, previous work by Theodosopoulos *et al*. demonstrated that the majority of the movement-related units responded to passive movements of a single joint or multiple joints of the same extremity, with only less than 5% of the units responsive to movement of multiple limbs^20^. This percentage perfectly matches our finding of 4.5% of the units related to both feet and hand movements, each performed during single-limb movement (Table 2, 3^rd^ data row, last column). Our results thus extend these findings from passive, non-rhythmic movements^20^ to also include voluntary, rhythmic active ones. More importantly, our research expands the type of movements to include ipsilateral movements and two-limb coordinated movements, actions not studied by Theodosopoulos *et al*.^20^.

The present results demonstrate that in Parkinson’s disease the STN combines information about the acting limbs with kinematic information about their movement. We found a population of units whose encoding of single-limb movements is apparently independent of the laterality of the performing limb: feet movements, independent of the actual performing foot, or hand movements, independent of the actual hand. The high proportion of this population within the recorded neuronal population cannot be explained by a random mixture of properties, indicating a representational abstraction of laterality in the STN. However, it seems that the STN may not “bind together” movements of the feet and hand of the same body side, as the proportion of neurons encoding this type of movements does match a random mixture of properties in the population. The proportion of the population of units that are both pure bipedal and pure bimanual also matches random mixture of these properties, lending further support to the lack of inherent “binding” of upper and lower extremities in the STN. The separate populations of pure bimanual and pure unimanual units explain why stimulation of the STN has a different effect on bimanual and unimanual movements^11^.

Our finding of a somatotopic organization of the STN expands the reported somatotopy in the monkey^24^,^26^,^27^ and human^28^,^29^ to the human wrist and ankle joints. We describe, in addition, a large sub-area with an overlap between feet and hand representations, where the same unit participates in control of both types of movement. This overlap zone may explain why no clear somatotopic map of upper- and lower-extremity receptive fields could be discerned in the sagittal plane^30^.

The large overlap may reflect changes in the somatotopy due to Parkinson’s disease. A similar finding has been reported in the monkey: neurons in the GPe/GPi and pallidonigral thalamus of the parkinsonian monkey lost their specificity to single contralateral joint and single preferred direction, and responded to multiple joints of the upper or lower extremities^31^,^32^. The reduced neuronal specificity also matches the responsiveness to multiple kinematic parameters: acceleration, angular velocity and orientation, in our results. Similarly, the high percentage of units holding kinematic information (93%) may also reflect an increase due to Parkinson’s disease, as was demonstrated in the parkinsonian monkey, where the proportion of responsive neurons quadrupled in GPi and nearly doubled in GPe with respect to the intact one^31^. In the monkey STN, treatment with MPTP did not significantly change the proportion of responding neurons or the latencies of neuronal responses of nonoscillatory STN neurons^33^ whereas in our results, large variations in time lags were observed in the human STN. However, the same study found that the average duration and magnitude of phasic responses to the application of flexion and extension torque pulses to the elbow tended to be increased in the parkinsonian monkey with respect to the intact one. These phenomena may be due to a compensation mechanism. The apparent discrepancies between the monkey and human STN may stem from inter-species differences, the different type of movement employed in each study (kinesthetic torque pulses vs. rhythmic voluntary movements), or differences in the compensatory mechanisms. In humans, somatosensory receptive fields were widened in patients with Parkinson’s disease compared with those with dystonia^34^.

The overlap is also in concordance with a certain interaction between the subthalamic representations of upper and lower extremities indirectly implied by the increased fMRI activation in patients with freezing of gait (lower extremity) during bilateral finger movements (upper extremity), compared to Parkinson’s disease patients who had no freezing and to healthy controls^35^.

Intra-operative single cell recordings provide a unique setup for studying the STN. To compare with the neurologically-normal STN, the inapplicability of the method to healthy humans limits comparisons to studies of other primates or lower species. The time constraints in the operating room also limit the possibility to record movement of additional body parts. Thus, some of the recorded neuronal activity may possibly be related to movement of unmonitored parts of the body.

Our results are an important step towards a more complete understanding of the pathophysiology of Parkinson’s disease and its compensatory mechanisms at the single neuron level. The findings may help facilitate more focused implantation of electrodes for deep brain stimulation or subthalamic lesioning therapies, targeting specific symptoms related to kinematics, and at more accurate locations within the STN. Thus, future implantation procedures may include intra-operative mapping the STN for representation of upper and lower limb movements for implantation in a specific subregion. For example, based on this mapping, tremor dominant patients exhibiting no gait disorders may be implanted in the more inferior-lateral part of the STN to avoid the subarea containing representation of gait. Whereas gripping force was shown to be decodable from the activity of subthalamic units in patients with Parkinson’s disease^1^, our findings indicate that care must be taken when attempting to generalize such reconstructions to individuals without Parkinson’s disease, for example for brain-machine interfaces for paralyzed persons. Interestingly, the decoded signal explained only 68% of the variability in hand gripping force^1^, which may possibly result from reduced specificity due to Parkinson’s disease. Nevertheless, decoding single unit activity^36^ in the STN has the potential to help restore functional control of hand and feet movements for new types of brain-machine interfaces designed to restore mobility, hand movements and/or their coordinated actions^37^.

## Methods

### Subjects and Electrophysiology

Participants in this study were ten patients with Parkinson’s disease who were undergoing implantation of electrodes for deep brain stimulation (3389, Medtronic Inc., Minneapolis, MN) when off medications for clinical reasons only. As part of the clinical procedure, two microelectrodes (Neuroprobe, Alpha-Omega, Nazareth, Israel), central and anterior, were temporarily implanted during surgery to locate the target brain area for implantation, based on single unit activity patterns. Bandpass filtered signals (0.3–3.0 kHz) from these microwires were recorded at 48 kHz using a 2-channel acquisition system (Neuroguide, Alpha-Omega, Nazareth, Israel). Spikes of individual cells were isolated based on their wavelet coefficients, the distribution of interspike intervals and the presence of a refractory period for the single units (that is, less than 1% of the inter-spike intervals are shorter than 3 ms) using superparamagnetic clustering (“WaveClus”)^38^,^39^. The study was approved by the Medical Institutional Review Board at the Tel Aviv Sourasky Medical Center and all patients provided written informed consent. All experiments were performed in accordance with relevant guidelines and regulations.

### Localization of Electrodes and Target Selection

MRI images co-registered to pre-operative CT scans were used with the BrainLab navigation planning software to locate the target and planned trajectory. The recording site, in Talairach coordinates^40^, was identified based on its distance from target along the implantation trajectory. These coordinates were then converted into the standard Montreal Neurological Institute (MNI) coordinates using the template adopted by the International Consortium for Brain Mapping (ICBM152; Statistical Parametric Mapping (SPM) toolbox version 12 for MATLAB, Mathworks, Inc.).

STN target area is initially identified based on standard AC-PC coordinates (3 mm posterior to MCP, 11 mm lateral to the midline, and 4 mm below AC-PC plane) and then further refined using indirect localization based on the red nucleus as an internal landmark. In most cases, the STN could also be directly identified on proton-density weighted images.

### Experimental Paradigms

Experiments were conducted intra-operatively (and thus, time was an important constraint), with the awake subject lying in a supine position, when the microelectrodes were inside the target area, the STN. Patients performed hand and feet tapping movements (defined as palmar flexion and extension at the wrist and dorsiflexion and plantar flexion at the ankle, respectively), one limb at a time, bipedally, or bimanually in an alternating pattern, each at three paces: slow, normal (i.e., self-selected) and fast. Subjects were instructed orally to start and stop each type of movement. All patients performed the task in the same order of movements. To keep movements voluntary, no external cuing was provided during the task. During the tasks, patients wore small, light, wireless movement-measurement devices (Opal monitors; APDM, Inc.) on the dorsa of their feet and hands, synchronized with the neuronal recording. The Opal monitors consist of accelerometers, gyroscopes and magnetometers. Accelerometer range is ±6 g, with a typical noise density of 1.3 mm/s^2^/√Hz. The gyroscopes have a range of: ±34.9 rad/s (typical noise density: 0.81 mrad/s/√Hz) in the X and Y axis, and ±26.8 rad/s (noise: 2.2 mrad/s/√Hz) in the Z axis. Magnetometers have a range of ±6 Gauss, with a typical noise density of 160 nT/√Hz. The sensors sample at 128 Hz.

### Statistical Analysis

The kinematic parameters and firing rate function were computed in the same 10 ms bins. The latter was then smoothed by convolution with a Gaussian function (SD = 50 ms) to obtain a continuous function. We examined time lags between the neuronal firing and actual movement in the range of (−1000) ms to 1000 ms, because the supplementary motor area, which sends inputs to the STN, can be active as early as 1200 ms before a new movement sequence is executed^41^,^42^. We modeled the smoothed firing rate function by a 12-fold cross-validated linear regression of each triaxial kinematic parameter: orientation, angular velocity and acceleration. The separate model for each parameter allows us to attribute the neuronal activity to a specific parameter. Orientation-based models also employed the sine and cosine of orientation in each axis. In addition, we constructed models employing all 3 kinematic parameters together. A model was constructed for each time lag independently. The time lag resulting in the maximal absolute value of the Pearson correlation coefficient between the model and the actual firing rate on a training set was selected^42^. The training set consisted of part of the tapping cycles performed by the patient. Reported correlation coefficients were calculated on an independent test set, composed of the tapping cycles that were not employed for constructing the linear model. Their p-values were Bonferroni-corrected (see “Significance of Correlation and Bonferroni Correction” below).

The entropy correlation coefficient^21^ is a measure of dependence between two variables, whose properties are direct consequences of the properties of the expected mutual information measure. However, the values of the entropy correlation coefficient range between 0 and 1, with 0 indicating full independence and 1 complete dependence. This information-based measure can quantify the dependence of two variables even if their relations are non-linear. To quantify the dependence between the firing rate and kinematics, their entropy correlation coefficient was computed for each tapping cycle. All entropy computations were bias-corrected using the Panzeri-Treves term^43^. Bonferroni-corrected *p*-values were computed using a nonparametric two-sided sign test for the null hypothesis that the distribution of the entropy correlation coefficients comes from a distribution whose median is zero. The median entropy correlation coefficient (over all tapping cycles) and the p-value for its being 0 were computed for each time lag (between (−1000) ms and 1000 ms, in 40 ms shifts). Among those time lags with a significant p-value (<0.05; adjusted by the false discovery rate), the one with the highest median entropy correlation coefficient is defined as the optimal time lag. Because the distribution of entropy correlation coefficients is not normal, the Mack-Skillings test^44^ served for nonparametric two-way unbalanced ANOVA.

### Significance of Correlation and Bonferroni Correction

We define that the correlation between the firing rate and its kinematics-based model is significant, when the probability *p* of getting a correlation as large as the observed value by random chance, when the true correlation is zero, is: *p* < 0.05. Reported *p*-values are Bonferroni-corrected with correction factor: 3 × 8 × 4 × 51 = 4,896 (3 paces (slow/normal/fast); 8 limb signals: (left foot, right foot, bipedal-left, bipedal-right, left hand, right hand, bimanual-left, bimanual-right); 4 types of kinematic models (acceleration, angular velocity, orientation, all parameters together); 51 time lags (between (−1000) ms and 1000 ms, in 40 ms shifts). Thus, the criterion is: p < 0.05/4,896 = 1.0E-5. A neuron was considered directly (linearly) encoding kinematics if the criteria for significance held for any of these tests, and in addition: *r*^2^ > 0.30 held, where *r*^2^ is the coefficient of determination between the model and actual firing rate.

### Random Mixture of Properties

To evaluate whether or not in a population of size n, a certain combination of properties may be due to a random mixture of properties in the population, we compared the actually observed proportion of units with the combined properties (n_obser_/n) with the proportion of units expected theoretically under the assumption of a random mixture of properties. Under this assumption, the expected proportion can be estimated by the product of the observed proportion of units with each property in the population: n_1_/n, n_2_/n. We therefore compare (n_obser_/n) with: (n_1_/n) * (n_2_/n). We tested whether the actually observed population and the theoretical one have the same proportions of observations using the Chi-square test of homogeneity between a population of size n with n_obser_ observations and a theoretical population of size n with n * (n_1_/n) * (n_2_/n) observations.

Thus, Table 2 (bottom row) suggests that the probability of encoding contralateral movements of feet or hands can be estimated by: P_contra_ = n_contra_/n = 37/89 = 0.42, and of encoding ipsilateral movements: P_ipsi_ = n_ipsi_/n = 35/89 = 0.39. The probability of observing units encoding both ipsi- and contra-lateral movements under the assumption of random mixture of properties is therefore: 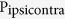 = P_ipsi_ * P_contra_ = 0.42 * 0.39 = 0.16. For a population of 89 recorded units, that should yield, on average, 89 * 0.16 = 14.24 units. Comparing the theoretical proportion with the observed 26 units, the Chi-square test of homogeneity yielded: p = 0.04, χ^2^ = 4.44, 1 degree of freedom, indicating that the observed proportion is significantly different from that drawn from a population with random mixture of properties.

## Additional Information

**How to cite this article**: Tankus, A. *et al*. Subthalamic Neurons Encode Both Single- and Multi-Limb Movements in Parkinson’s Disease Patients. *Sci. Rep.* **7**, 42467; doi: 10.1038/srep42467 (2017).

**Publisher's note:** Springer Nature remains neutral with regard to jurisdictional claims in published maps and institutional affiliations.

## Figures and Tables

**Table 1:** Patient clinical evaluation and demographics.

	Gender	Age	Dominant Hand	Disease Duration	Hoehn and Yahr	Activities of Daily Living (UPDRS II)		Motor Examination (UPDRS III)		Total UPDRS Score	
						OFF	ON	OFF	ON	OFF	ON
P1	M	45	R	15	3	19*		27*		60*	
P2	M	41	R	8	2	Not Available					
P3	M	70	R	17	3	3	0	15	6	19	7
P4	M	66	R	9	2.5	15	8	33	26	50	36
P5	M	61	R	6	3	9	1	15	3	28	8
P6	M	59	R	8	2	17	14	39	23	59	40
P7	M	57	R	10	2	10	2	17	15	27	17
P8	M	47	R	7	2.5	11	7	29	16	47	30
P9	F	74	R	17	2.5		23*		17*		57*
P10	F	72	R	14	3		11*		16*		54*
Mean (SD)	—	59.2 (11.7)	—	11.1 (4.2)	2.55 (0.44)	10.8 (4.9)	5.3 (5.4)	24.7 (10.4)	14.8 (9.1)	38.3 (15.8)	23.0 (14.3)

*MDS-UPDRS. Mean and standard deviation (SD) of UPDRS scores excludes patients who performed the MDS-UPDRS version. MDS = Movement Disorders Society. UPDRS = Unified Parkinson’s Disease Rating Scale (higher scores indicate worse symptoms).

**Table 2:** Percentage (of the total 89 recorded units) and number (in parentheses) of pure unipedal or pure unimanual units according to the laterality of the limb performing the movement they relate to.

	Single Limb Movements			
	Contralateral	Ipsilateral	Both contra- and ipsi-lateral movements (but single limb at a time)	Total
Foot	31% (28)	26% (23)	18% (16)	39% (35)
Hand	15% (13)	15% (13)	12% (11)	17% (15)
Same unit is related to both feet and hand movements	4.5% (4)	1.1% (1)	1.1% (1)	4.5% (4)
Total	42% (37)	39% (35)	29% (26)	51% (45)

## References

[b1] Patil P. G. M. D., Carmena J. M., Nicolelis M. A. L. M. D. & Turner D. A. Ensemble recordings of human subcortical neurons as a source of motor control signals for a brain-machine interface. Neurosurg. 55, 27–38 (2004). 15214971

[b2] Hanson T. L., Fuller A. M., Lebedev M. A., Turner D. A. & Nicolelis M. A. L. Subcortical Neuronal Ensembles: An Analysis of Motor Task Association, Tremor, Oscillations, and Synchrony in Human Patients. J. Neurosci. 32, 8620–8632 (2012). 22723703 10.1523/JNEUROSCI.0750-12.2012 PMC3502028

[b3] Hutchison W. D. . Neurophysiological identification of the subthalamic nucleus in surgery for Parkinson’s disease. Ann. Neurol. 44, 622–628 (1998). 9778260 10.1002/ana.410440407

[b4] Wu T., Wang L., Hallett M., Li K. & Chan P. Neural correlates of bimanual anti-phase and in-phase movements in Parkinson’s disease. Brain 133, 2394–2409 (2010). 20566485 10.1093/brain/awq151 PMC3139934

[b5] Piper M., Abrams G. M. & Marks W. J. Jr. Deep brain stimulation for the treatment of Parkinson’s disease: Overview and impact on gait and mobility. NeuroRehabilitation 20, 223–232 (2005). 16340102

[b6] Hausdorff J. M., Gruendlinger L., Scollins L., O’Herron S. & Tarsy D. Deep brain stimulation effects on gait variability in Parkinson’s disease. Mov. Disord. Off. J. Mov. Disord. Soc. 24, 1688–1692 (2009). 10.1002/mds.22554 19554569

[b7] Lau B. . The integrative role of the pedunculopontine nucleus in human gait. Brain 138, 1284–1296 (2015). 25765327 10.1093/brain/awv047 PMC5963406

[b8] Georgopoulos A. P., DeLong M. R. & Crutcher M. D. Relations between parameters of step-tracking movements and single cell discharge in the globus pallidus and subthalamic nucleus of the behaving monkey. J. Neurosci. 3, 1586–1598 (1983). 6875658 10.1523/JNEUROSCI.03-08-01586.1983 PMC6564524

[b9] DeLong M. R., Crutcher M. D. & Georgopoulos A. P. Primate globus pallidus and subthalamic nucleus: functional organization. J. Neurophysiol. 53, 530–543 (1985). 3981228 10.1152/jn.1985.53.2.530

[b10] Hikosaka O. Basal ganglia–possible role in motor coordination and learning. Curr. Opin. Neurobiol. 1, 638–643 (1991). 1822310 10.1016/s0959-4388(05)80042-x

[b11] Alberts J. L., Okun M. S. & Vitek J. L. The persistent effects of unilateral pallidal and subthalamic deep brain stimulation on force control in advanced Parkinson’s patients. Parkinsonism Relat. Disord. 14, 481–488 (2008). 18342565 10.1016/j.parkreldis.2007.11.014 PMC2605295

[b12] Alberts J. L. . Bilateral subthalamic stimulation impairs cognitive–motor performance in Parkinson’s disease patients. Brain 131, 3348–3360 (2008). 18842609 10.1093/brain/awn238 PMC2639204

[b13] Gorniak S. L., McIntyre C. C. & Alberts J. L. Bimanual Force Coordination in Parkinson’s Disease Patients with Bilateral Subthalamic Deep Brain Stimulation. PLoS One 8 (2013). 10.1371/journal.pone.0078934 PMC3823934 24244388

[b14] Fasano A. . Modulation of gait coordination by subthalamic stimulation improves freezing of gait. Mov. Disord. 26, 844–851 (2011). 21370271 10.1002/mds.23583

[b15] Schettino L. F. . Deep brain stimulation of the subthalamic nucleus facilitates coordination of hand preshaping in Parkinson’s disease. Int. J. Neurosci. 119, 1905–1924 (2009). 19922392 10.1080/00207450903245296

[b16] Johnsen E. L., Mogensen P. H., Sunde N. A. & Østergaard K. Improved asymmetry of gait in Parkinson’s disease with DBS: gait and postural instability in Parkinson’s disease treated with bilateral deep brain stimulation in the subthalamic nucleus. Mov. Disord. Off. J. Mov. Disord. Soc. 24, 590–597 (2009). 10.1002/mds.22419 19097189

[b17] Tinakoua A. . The impact of combined administration of paraquat and maneb on motor and non-motor functions in the rat. Neuroscience 311, 118–129 (2015). 26477982 10.1016/j.neuroscience.2015.10.021

[b18] Leunissen I. . Disturbed cortico-subcortical interactions during motor task switching in traumatic brain injury. Hum. Brain Mapp. 34, 1254–1271 (2013). 22287257 10.1002/hbm.21508 PMC6870055

[b19] Coxon J. P. . Reduced Basal Ganglia Function When Elderly Switch between Coordinated Movement Patterns. Cereb. Cortex 20, 2368–2379 (2010). 20080932 10.1093/cercor/bhp306

[b20] Theodosopoulos P. V., Marks W. J., Christine C. & Starr P. A. Locations of movement-related cells in the human subthalamic nucleus in Parkinson’s disease. Mov. Disord. 18, 791–798 (2003). 12815658 10.1002/mds.10446

[b21] Virtanen I. In Essays in Management Studies 15–18 (1983).

[b22] Ponsen M. M. . Bimanual coordination dysfunction in early, untreated Parkinson’s disease. Parkinsonism Relat. Disord. 12, 246–252 (2006). 16621659 10.1016/j.parkreldis.2006.01.006

[b23] Plotnik M., Giladi N., Dagan Y. & Hausdorff J. M. Postural instability and fall risk in Parkinson’s disease: impaired dual tasking, pacing, and bilateral coordination of gait during the ‘ON’ medication state. Exp. Brain Res. 210, 529–538 (2011). 21279632 10.1007/s00221-011-2551-0

[b24] Wichmann T., Bergman H. & DeLong M. R. The primate subthalamic nucleus. I. Functional properties in intact animals. J. Neurophysiol. 72, 494–506 (1994). 7983514 10.1152/jn.1994.72.2.494

[b25] Sheridan M. R., Flowers K. A. & Hurrell J. Programming and Execution of Movement in Parkinson’s Disease. Brain 110, 1247–1271 (1987). 3676700 10.1093/brain/110.5.1247

[b26] Nambu A., Takada M., Inase M. & Tokuno H. Dual somatotopical representations in the primate subthalamic nucleus: evidence for ordered but reversed body-map transformations from the primary motor cortex and the supplementary motor area. J. Neurosci. 16, 2671–2683 (1996). 8786443 10.1523/JNEUROSCI.16-08-02671.1996 PMC6578767

[b27] Nambu A. Somatotopic Organization of the Primate Basal Ganglia. Front. Neuroanat. 5 (2011). 10.3389/fnana.2011.00026 PMC3082737 21541304

[b28] Rodriguez-Oroz M. C. . The subthalamic nucleus in Parkinson’s disease: somatotopic organization and physiological characteristics. Brain 124, 1777–1790 (2001). 11522580 10.1093/brain/124.9.1777

[b29] Romanelli P., Esposito V., Schaal D. W. & Heit G. Somatotopy in the basal ganglia: experimental and clinical evidence for segregated sensorimotor channels. Brain Res. Rev. 48, 112–128 (2005). 15708631 10.1016/j.brainresrev.2004.09.008

[b30] Abosch A., Hutchison W. D., Saint-Cyr J. A., Dostrovsky J. O. & Lozano A. M. Movement-related neurons of the subthalamic nucleus in patients with Parkinson disease. J. Neurosurg. 97, 1167–1172 (2002). 12450039 10.3171/jns.2002.97.5.1167

[b31] Filion M., Tremblay L. & Bédard P. J. Abnormal influences of passive limb movement on the activity of globus pallidus neurons in parkinsonian monkeys. Brain Res. 444, 165–176 (1988). 3359286 10.1016/0006-8993(88)90924-9

[b32] Pessiglione M. . Thalamic Neuronal Activity in Dopamine-Depleted Primates: Evidence for a Loss of Functional Segregation within Basal Ganglia Circuits. J. Neurosci. 25, 1523–1531 (2005). 15703406 10.1523/JNEUROSCI.4056-04.2005 PMC6725984

[b33] Bergman H., Wichmann T., Karmon B. & DeLong M. R. The primate subthalamic nucleus. II. Neuronal activity in the MPTP model of parkinsonism. J. Neurophysiol. 72, 507–520 (1994). 7983515 10.1152/jn.1994.72.2.507

[b34] Schrock L. E., Ostrem J. L., Turner R. S., Shimamoto S. A. & Starr P. A. The Subthalamic Nucleus in Primary Dystonia: Single-Unit Discharge Characteristics. J. Neurophysiol. 102, 3740–3752 (2009). 19846625 10.1152/jn.00544.2009 PMC4073906

[b35] Vercruysse S. . The Neural Correlates of Upper Limb Motor Blocks in Parkinson’s Disease and Their Relation to Freezing of Gait. Cereb. Cortex 24, 3154–3166 (2014). 23861319 10.1093/cercor/bht170

[b36] Tankus A., Fried I. & Shoham S. Sparse decoding of multiple spike trains for brain–machine interfaces. J. Neural Eng. 9, 54001 (2012). 10.1088/1741-2560/9/5/054001 PMC4445936 22954906

[b37] Tankus A., Fried I. & Shoham S. Cognitive-motor brain–machine interfaces. J. Physiol.-Paris 108, 38–44 (2014). 23774120 10.1016/j.jphysparis.2013.05.005 PMC4424044

[b38] Quian-Quiroga R., Nadasdy Z. & Ben-Shaul Y. Unsupervised spike detection and sorting with wavelets and superparamagnetic clustering. Neural Comput. 16, 1661–1687 (2004). 15228749 10.1162/089976604774201631

[b39] Tankus A., Yeshurun Y. & Fried I. An automatic measure for classifying clusters of suspected spikes into single cells versus multiunits. J. Neural Eng. 6, 56001 (2009). 10.1088/1741-2560/6/5/056001 PMC2837589 19667458

[b40] Talairach J. & Tournoux P. Co-planar Stereotaxic Atlas of the Human Brain: 3-Dimensional Proportional System - an Approach to Cerebral Imaging (Thieme Medical Publishers, 1988).

[b41] Lang W. . Three-dimensional localization of SMA activity preceding voluntary movement. A study of electric and magnetic fields in a patient with infarction of the right supplementary motor area. Exp. Brain Res. 87, 688–695 (1991). 1783038 10.1007/BF00227095

[b42] Tankus A., Yeshurun Y., Flash T. & Fried I. Encoding of Speed and Direction of Movement in the Human Supplementary Motor Area. J. Neurosurg. 110, 1304–1316 (2009). 19231930 10.3171/2008.10.JNS08466 PMC2837583

[b43] Panzeri S. & Treves A. Analytical estimates of limited sampling biases in different information measures. Netw. Comput. Neural Syst. 7, 87–107 (1996). 10.1080/0954898X.1996.11978656 29480146

[b44] Mack G. A. & Skillings J. H. A Friedman-Type Rank Test for Main Effects in a Two-Factor ANOVA. J. Am. Stat. Assoc. 75, 947 (1980).

[b45] Keuken M. C. . Quantifying inter-individual anatomical variability in the subcortex using 7T structural MRI. NeuroImage 94, 40–46 (2014). 24650599 10.1016/j.neuroimage.2014.03.032

